# Mitochondria in the Crossfire: Cardiac Vulnerability During Chemotherapy

**DOI:** 10.3390/cancers18040674

**Published:** 2026-02-19

**Authors:** Teresa Pasqua, Maria Erika D’Alessandro, Nicola Amodio

**Affiliations:** 1 Department of Health Science, University Magna Graecia of Catanzaro, 88100 Catanzaro, Italy; mariaerika.dalessandro@studenti.unicz.it; 2 Department of Experimental and Clinical Medicine, University Magna Graecia of Catanzaro, 88100 Catanzaro, Italy; amodio@unicz.it

Despite substantial advances in oncology, chemotherapy-associated adverse outcomes, most notably cardiotoxicity, remain a significant threat to patient quality of life and long-term survival. Anthracyclines (ANTs) are highly effective and widely used anticancer agents in the treatment of both solid and hematological malignancies; however, their clinical utility is constrained by a well-recognized risk of dose-dependent and often irreversible cardiac injury [1,2,3]. This toxicity encompasses a broad temporal spectrum, ranging from acute myocardial injury to early- and late-onset chronic heart failure, which may manifest years after the completion of chemotherapy [4,5]. Notwithstanding decades of investigation, the molecular mechanisms underpinning ANT-induced damage remain incompletely understood and, in several aspects, controversial.

Traditionally, ANTs’ cardiotoxicity has been explained by a combination of DNA damage and oxidative stress. A major conceptual breakthrough was provided by the identification of Topoisomerase IIβ (Top2β) as a critical mediator of cardiac injury. Unlike proliferating tumor cells, which predominantly express the Top2α isoform, adult cardiomyocytes express, also within mitochondria, Top2β [6]. ANTs bind Top2β producing DNA double-strand breaks, transcriptional dysregulation, and cardiomyocyte damage that ultimately lead to the development of cardiomyopathy [7]. Importantly, this mechanism aligns with the post-mitotic nature of cardiomyocytes, suggesting that mitochondrial and nuclear DNA damage, rather than impaired cell division, may represent a primary driver of disease. However, ANTs exert profound effects on mitochondrial structure and function that go beyond the Top2β-mediated mechanism.

Doxorubicin (Doxo), a representative ANT, accumulates within mitochondria through its high affinity for cardiolipin, a phospholipid enriched in the inner mitochondrial membrane. This interaction promotes the drug mitochondrial retention and the subsequent inhibition of Complex I and II of the Electron Transport Chain, triggering a “futile redox cycling”, characterized by exacerbated reactive oxygen species (ROS) production and mitochondrial iron accumulation [8,9]. Since Top2β silencing attenuates ROS production only partially, the persisting oxidative stress indicates that Top2β-independent mitochondrial mechanisms substantially contribute to cardiotoxicity [7].

Nevertheless, the attempts to use traditional antioxidants, such as dexrazoxane, in managing cardiotoxicity have yielded disappointing clinical results, especially in terms of broad/non-selective action. The limited efficacy observed is largely due to their inability to selectively accumulate within the mitochondrial matrix, where the bulk of ANTs-induced damage occurs [10,11,12]. This deadlock has shifted the attention toward Mitochondria-Targeted Antioxidants (MTAs), able to bind positively charged groups, exploit the negative charge of mitochondrial membrane, and concentrate within these organelles. MTAs not only scavenge ROS but also preserve mitochondrial membrane integrity and modulate mitochondrial dynamics, positioning them as promising cardioprotective candidates [13,14].

In this complex scenario and according to these observations, the focus of the field has moved to mitochondrial quality control mechanisms and mitochondrial dynamics as a central determinant of cardiomyocyte survival during ANTs exposure. Indeed, mitochondrial homeostasis is maintained through the coordinated regulation of mitochondrial biogenesis, dynamics (fusion and fission), and selective mitochondrial autophagy (mitophagy) [15,16,17]. Disruption of any of these processes can compromise cardiac metabolism and contractile function, rendering the heart particularly vulnerable to ANTs-induced stress [18].

Within this conceptual framework, the recent study by Díaz-Guerra and colleagues [19] represents a significant advance. For the first time, they paved the way to recognize the temporal stage as a potential therapeutic topic in cardiotoxicity development. In fact, rather than treating ANTs’ cardiotoxicity as a static condition, the authors proposed and experimentally validated the idea that it is a dynamic and stage-dependent mitochondrial disease. This perspective challenged the prevailing reliance on cross-sectional analyses and introduced time as a critical variable in both mechanistic understanding and therapeutic intervention. Using a chronic slow-progression model of ANTs-induced cardiac dysfunction, Díaz-Guerra et al. performed a comprehensive longitudinal assessment of cardiac metabolism, mitochondrial ultrastructure, and quality control pathways across distinct stages of the disease development. The authors employed a chronic mouse model providing weekly intraperitoneal injections of Doxo (5 mg/kg) for 5 weeks, with extended monitoring to 15 weeks to mimic clinical long-term toxicity. This approach allowed them to capture adaptive and maladaptive mitochondrial responses that are obscured in single time-point studies. Their findings revealed that mitochondrial remodeling during ANTs exposure is not uniform but evolves in a highly coordinated and temporally regulated manner. One of the most striking observations from this work was the identification of an early adaptive phase characterized by enhanced mitophagy. In the very preliminary stages of ANTs exposure, cardiomyocytes activate mitophagy pathways as a compensatory mechanism aimed at removing damaged mitochondria and preserving overall mitochondrial integrity. During this phase, mitochondrial structure is relatively preserved, suggesting that efficient quality control temporarily buffers the heart against ANTs-induced injury. This finding agrees with the concept that mitophagy serves as a protective mechanism in stressed cardiomyocytes, maintaining energy production and limiting ROS accumulation. However, as ANTs exposure persists, mitophagy progressively declines, coinciding with marked mitochondrial fragmentation and disruption of the mitochondrial network. This transition from a protective to a maladaptive state represents a critical inflection point in disease progression. Excessive mitochondrial fission, together with the impaired clearance of dysfunctional organelles, promotes energetic failure and cardiomyocyte dysfunction, ultimately contributing to the development of overt cardiomyopathy. Conversely, cardiac metabolism showed a marked decline from the early stage as a possible consequence of ultrastructural damage in the inner mitochondrial membrane. Thus, metabolic impairment precedes structural mitochondrial collapse suggesting that disruption of respiratory efficiency is an initiating event rather than a late consequence of cardiotoxicity. These results reinforce the hypothesis that metabolic remodeling is a sensitive and early indicator of ANTs-induced cardiac injury. Taken together, the observations by Diaz-Guerra et al. [19] seamlessly integrate the existing notions on mitochondrial biogenesis and dynamics. Indeed, it is known that ANTs alter PGC-1α-dependent transcriptional programs that control mitochondrial biogenesis, in part by Top2β-mediated transcriptional repression, p53 activation, and downregulation of Sirtuin-1 [20,21,22]. Surprisingly, Diaz-Guerra and collaborators reported compensatory changes in these pathways during the early disease stages, followed by their breakdown in cardiotoxicity progression. This temporal pattern suggests that therapeutic strategies aimed at enhancing mitochondrial biogenesis or preserving metabolic capacity may be most effective if applied early, before irreversible mitochondrial damage occurs. From a translational point of view, by defining ANTs’ cardiotoxicity as a temporally evolving mitochondrial disorder, Diaz-Guerra et al. provided intriguing implications. They suggested that a stage-specific therapy could overcome the limitations imposed by “one-size-fits-all” cardioprotective interventions. Accordingly, an early enhancement of mitophagy and metabolism, followed by a later modulation of mitochondrial dynamics, may confer increased cardioprotection. This paradigm may also help explain the inconsistent outcomes of previous cardioprotective trials, in which therapeutic timing was not aligned with the underlying biology of disease progression. The longitudinal approach of this work establishes time-dependent mitochondrial remodeling as a central determinant of cardiac outcome, paving new ways for the development of precisely timed, mitochondria-targeted cardioprotective strategies.

In summary, the evolving landscape of cardio-oncology suggests that mitochondria are not merely collateral victims of chemotherapy but rather the “ground zero”, where the trajectory of heart failure is determined. Moreover, the recognition of ANTs-induced cardiotoxicity as a dynamic and stage-dependent mitochondrial disorder, rather than a linear static event, marks a pivotal shift in therapeutic philosophy. Consequently, innovative therapeutic avenues are evolving beyond simple ROS scavenging and beyond other conventional cardioprotective regimens, such as ACE inhibitors, β-blockers, and SGLT2 inhibitors, whose long-term efficacy in this specific context remains under rigorous investigation. Integrating these novel mitochondrial insights into clinical practice may ensure that the life-saving success of modern chemotherapy is not overshadowed by the long-term burden of heart disease.
